# Supplementary material for: Conferring High IAA Productivity on Low-IAA-Producing Organisms with PonAAS2, an Aromatic Aldehyde Synthase of a Galling Sawfly, and Identification of Its Inhibitor
Source: Insects. 2023 Jul 2;14(7):598. doi: 10.3390/insects14070598 (PMC10380194; doi:10.3390/insects14070598)
Supplement: Supplementary file 1 [file insects-14-00598-s001.zip › Table S1.pdf]

Table S1. Sequences of promoters used in this study.

GFP_fw	5'-CCGGATCCATGAGTAAAGGAGAAGAACT (*Bam*HI)
GFP_rv	5'-GTCGACAACACGCGTTCATTTGTATAGTTCGTCCATGC (*Sal*I, *Mlu*I)
unc-54-ter_fw	5'-ACGCGTGTCCAATTACTCTTCAACATC (*Mlu*I)
unc-54-ter_rv	5'-GTCGACAACGGCCACGGTGGCCAAACAGTTATGTTTGGTA (SalI, SfiI)
cdc-42-pro_fw	5'-CCGGTACCCGATTCTAGAGGAGGTCGTCG (KpnI)
cdc-42-pro_rv	5'-CCGGATCCCAACCCCTAGGTTCGCCTGAAAAAAAAAATG (BamHI, BlnI)
