# Supplementary material for: Conferring High IAA Productivity on Low-IAA-Producing Organisms with PonAAS2, an Aromatic Aldehyde Synthase of a Galling Sawfly, and Identification of Its Inhibitor
Source: Insects. 2023 Jul 2;14(7):598. doi: 10.3390/insects14070598 (PMC10380194; doi:10.3390/insects14070598)
Supplement: Supplementary file 1 [file insects-14-00598-s001.zip › Table S2.pdf]

Table S2. Information of PonAAS2 inhibitors found by the initial screening.

	molecular formula	molecular weight	concentration (μM)[1]	
HTS09643	$C_{15}H_{11}NO_6S$	333.3	120.0	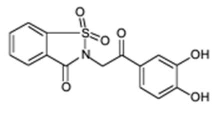
HTS09784	$C_{13}H_{20}N_6O$	276.3	144.7	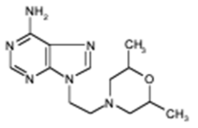
HTS10037	$C_{14}H_{17}NO_3$	247.3	161.7	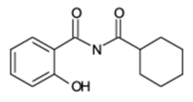
HTS10442	$C_{12}H_9NO_4$	231.2	173.0	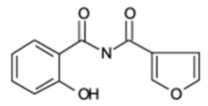
HTS10550	$C_{16}H_{13}N_3O_2$	279.3	143.2	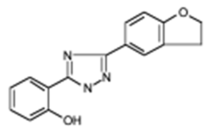
HTS11483	$C_{11}H_{10}ClN_3$	219.7	182.1	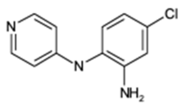
HTS12813	$C_{12}H_9N_3OS$	243.3	164.4	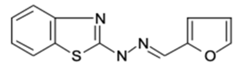
HTS12892	$C_{11}H_9NO_2S$	219.3	182.4	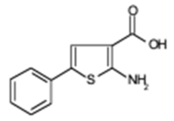
JA00082	$C_6H_6N_4O_2$	166.1	240.8	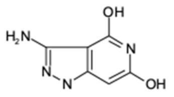
JFD03671	$C_{16}H_{11}N_3O$	261.3	153.1	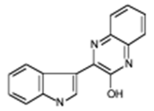
JFD03939	C9H6INO4S	351.1	113.9	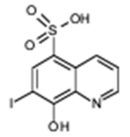

[1] Concentrations of compounds in the reaction used in the first screening were shown as μM.
