# Supplementary figures and images for: Conferring High IAA Productivity on Low-IAA-Producing Organisms with PonAAS2, an Aromatic Aldehyde Synthase of a Galling Sawfly, and Identification of Its Inhibitor
Source: Insects. 2023 Jul 2;14(7):598. doi: 10.3390/insects14070598 (PMC10380194; doi:10.3390/insects14070598)

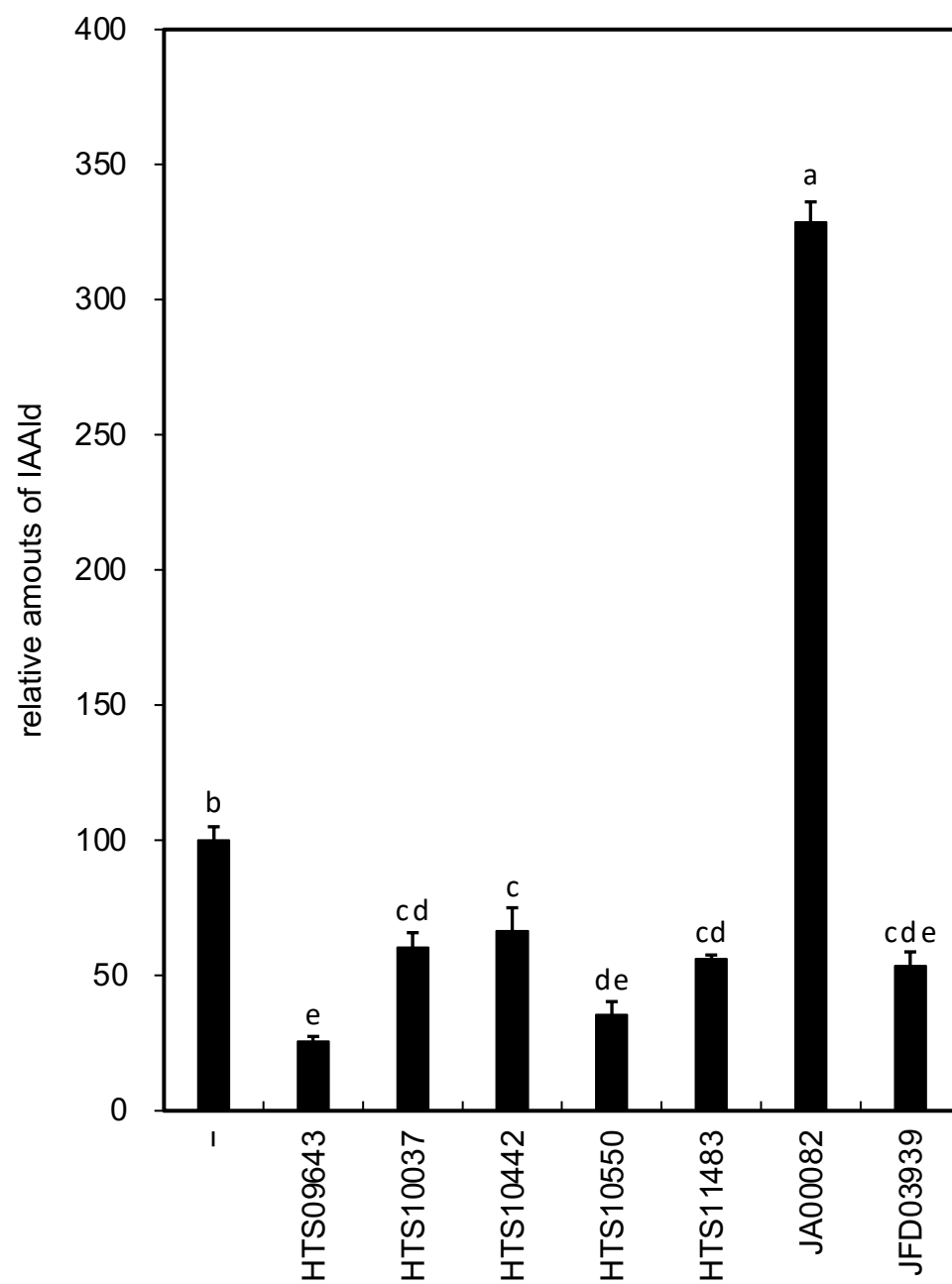

Supplement: Supplementary file 1 [file insects-14-00598-s001.zip › Figure S1.pdf]

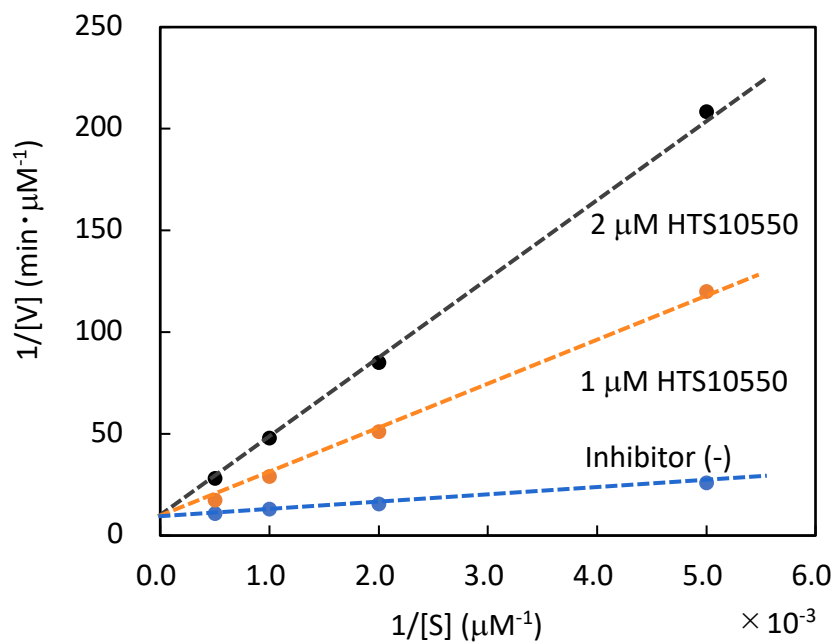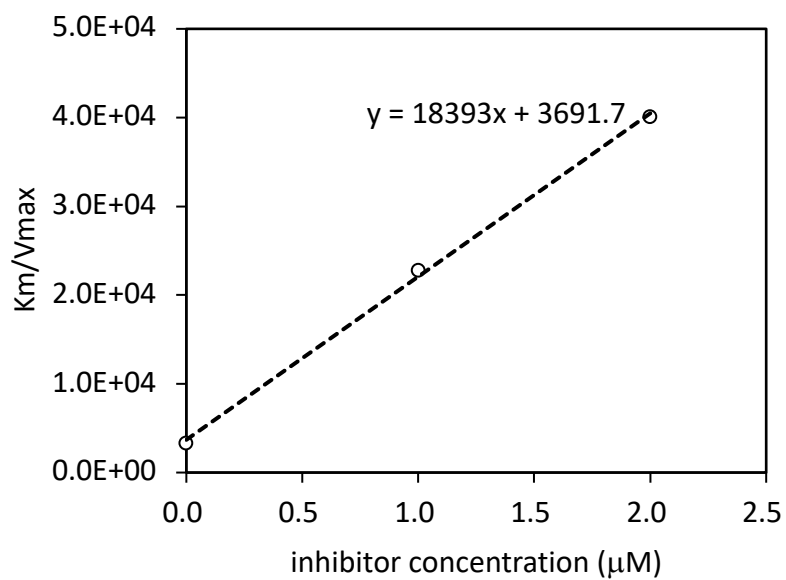

Supplement: Supplementary file 1 [file insects-14-00598-s001.zip › Figure S2.pdf]
